# INFLUENCE OF MINIMALLY INVASIVE LAPAROSCOPIC EXPERIENCE SKILLS ON ROBOTIC SURGERY DEXTERITY

**DOI:** 10.1590/0102-672020210003e1604

**Published:** 2021-12-17

**Authors:** Marcos BELOTTO, Larissa COUTINHO, Adhemar M PACHECO-JR, Anuar I. MITRE, Eduardo Antunes da FONSECA

**Affiliations:** 1 Department of Surgery, Pancreas Division, Santa Casa de São Paulo, São Paulo, SP, Brazil; 2 University of Taubate, Taubate, SP, Brazil; 3 Sirio-Libanes Hospital, São Paulo, Brazil

**Keywords:** Robotic, Laparoscopy, Motor skills, High fidelity simulation training, Robótica, Laparoscopia, Habilidades motoras, Treinamento de simulação de alta fidelidade

## Abstract

**Background:**

It is unclear if there is a natural transition from laparoscopic to robotic surgery with transfer of abilities.

**Aim:**

To measure the performance and learning of basic robotic tasks in a simulator of individuals with different surgical background.

**Methods:**

Three groups were tested for robotic dexterity**:** a) experts in laparoscopic surgery (n=6); b) experts in open surgery (n=6); and c) non-medical subjects (n=4). All individuals were aged between 40-50 years. Five repetitions of four different simulated tasks were performed: spatial vision, bimanual coordination, hand-foot-eye coordination and motor skill.

**Results:**

Experts in laparoscopic surgery performed similar to non-medical individuals and better than experts in open surgery in three out of four tasks. All groups improved performance with repetition.

**Conclusion:**

Experts in laparoscopic surgery performed better than other groups but almost equally to non-medical individuals. Experts in open surgery had worst results. All groups improved performance with repetition.

## RESUMO

**Racional:**

É incerto se há transferência natural de habilidades da cirurgia laparoscópica para a robótica.

**Objetivo:**

Avaliar o desempenho e aprendizado de tarefas em plataforma robótica simulada em indivíduos com diferentes conhecimentos em cirurgia.

**Método:**

Três grupos de indivíduos foram testados quanto à habilidade robótica: a) especialistas em cirurgia laparoscópica (n=6); b) especialista em cirurgia convencional (n=6); e c) indivíduos não médicos. A idade variou em todo grupo entre 40-50 anos. Cinco repetições de quatro tarefas simuladas foram realizadas: visão espacial, coordenação bimanual, coordenação mão-pé-olho e destreza manual.

**Resultados:**

Especialistas em cirurgia laparoscópica tiveram desempenho semelhante aos indivíduos não médicos e melhor que os especialistas em cirurgia convencional em três das quatro tarefas. Todos os grupos melhoraram desempenho com repetições ***.***

**Conclusão:**

Especialistas em cirurgia laparoscópica desempenharam melhor que os outros grupos, mas quase igualitariamente aos indivíduos não médicos. Especialista em cirurgia convencional apresentaram os piores resultados. Todos os grupos melhoraram com as repetições.

## INTRODUCTION

Robotic surgery may be considered by some a natural evolution of laparoscopic surgery; however, there are noteworthy differences between these two minimally invasive techniques^20^. These dissimilarities may lead to the assumption that there is no transference of laparoscopic abilities to the robotic platform but a need to abandon some previous aptitudes to learn new skills^33^. 

Robotic skills can be adequately trained and evaluated by realist simulators^19^. Previous studies compared robotic skills in individuals with different laparoscopic backgrounds to show in its majority similar results for experts and novices^26, 33^. The similarity of performance suggests a human natural ability to manipulate robotic instruments, i.e., robotic platform is apt to capture all-natural movements. These studies, however, compared individuals from different generations (usually medical students or residents vs. senior surgeons) bringing advantages to the neophytes more used to technology and videogames whose abilities are transferable to simulators^28^. 

 We believe that a protocol to evaluate if there is a natural transition of laparoscopic skills to robotic platform or a better ability of surgical robots to capture human natural movements must compare surgeons with different degrees of laparoscopic experience and individuals unfamiliar to surgical techniques and surgical simulation all from the same generation. 

This study aims to measure the performance and learning of basic robotic tasks in a simulator of individuals with different laparoscopic background and non-medical individuals. 

## METHOD

The protocol was approved by local IRB and informed consent was obtained from all individuals.

### Population

Three groups of individuals from 40-50 years of age, without previous robotic surgery experience were recruited: A) group 1 (n=6, 100% males), age 45 (41-47) years), experts in laparoscopic surgery, over five years and over 100 complex procedures, all gastrointestinal surgeons; B) group 2 (n=6, 83% males), age 44 (43-44) years), experts in open surgery, over five years, over 100 complex procedures, less than 10 simple laparoscopic procedures per year, no performance of complex laparoscopic procedures, all gastrointestinal surgeons; C) group 3 (n=4, 50% males), age 42 (41-45) years, two lawyers, one publicist, one financial analyst), individuals whose professions are apart from healthcare and robotic platforms. 

### Simulator

A realistic robotic simulator was used to assess robotic abilities (Mimic, Intuitive Surgery, Sunnyvale). The simulator has two manual joysticks and seven pedal switches. Individuals adopt a position similar to the real robotic platform commanding simulated scenarios depicting preloaded basic tasks. Performance was measured using a score from 0 to 100 considering time to perform the task, instruments collision, manual dexterity, force applied to the instruments and economy of movement. 

**FIGURE 1:**
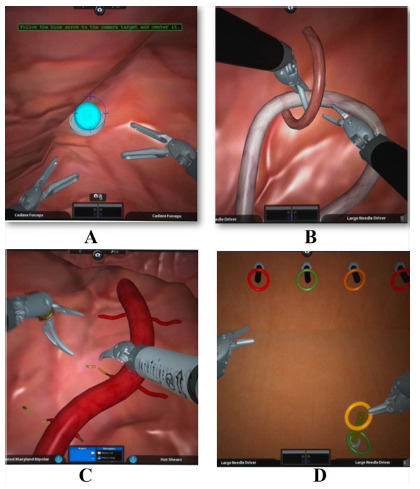
Simulated tasks at the robotic platform: A) camera targeting; B) ring walk; C) energy switching; D) pick & place

Individuals were instructed to watch an educative video resident in the system and perform five repetitions of four basic tasks: 1) “camera targeting*”:* individuals were asked to step on the camera pedal and focus on the targets using the joysticks; the task evaluated spatial vision**;** 2) “ring walk”: participants were asked to transfer rings from one point to the other using both hands; this task evaluated bimanual skills; 3) “energy switching”: individuals were asked to apply different coagulating energies in random targets using two different pedals, this task evaluated hand-feet-eyes coordination; and 4) “pick & place”: individuals were asked to sort objects according to colors; this task evaluated motor skills (Figure 1). 

### Statistical analysis

Variables were expressed as median (quartile 25-75), p<0.05 was set as significant. Mann-Whitney and Kruskall-Whallis were used to compared medians; Fisher test was used to compare proportions and Durbin-Watson test to evaluate temporal tendency. 

## RESULTS

There were no statistical differences among groups on gender (p=0.2) and age (p=0.9). All individuals completed the tasks. 

**FIGURE 2:**
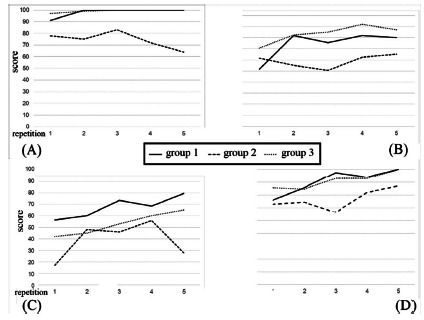
Temporal tendency of performance scores for basic simulated robotic skills: A) camera targeting; B) ring walk; C) energy switching; D) pick & place

Performance scores for the three groups are depicted in Table 1. Experts in laparoscopic surgery performed similar to non-medical individuals and better than experts in open surgery in three out of four tasks. Temporal tendency of performance scores is expressed in Figure 2. All groups improved performance with repetition.

**TABLE 1:** Performance scores for simulated basic robotic tasks

Task	Group 1 (surgeons experienced in laparoscopic surgery)	Group 2 (surgeons experienced in open surgery)	Group 3 (non-medical - controls)	Comparison among groups
Camera targeting	98 (69-100) [53-100]	73 (47-96) [13-100]	97 (72-98) [34-100]	1x2 p<0.001 * 1x3 p=0.2 2x3 p=0.02 *
Ring walk	78 (42-88) [19-96]	61 (38-67) [10-95]	85 (74-91) [30-96]	1x2 p=0.08 1x3 p=0.1 2x3 p<0.001 *
Energy switching	69 (47-810 [24-91]	44 (19-56) [0-97]	52 (36-64) [27-84]	1x2 p<0.001 * 1x3 p=0.02 * 2x3 p=0.09
Pick & place	81 (70-90) [48-94]	65 (56-76) [35-93]	83 (74-88) [57-94]	1x2 p<0.001 * 1x3 p=0.8 2x3 p<0.001 *

Variables are expressed as median (quartile 25 - 75); *=statistical significant

## DISCUSSION

### Differences between laparoscopic and robotic learning

There are pros and cons associated to robotic surgery in comparison to laparoscopic surgery; however, most of them are directed towards the operator with an indirect benefit to the patient only. This study considers that are technical differences between these two types of minimally invasive approaches, not only for the performance of the operation such as the process of docking, neither the 3-D vision or articulated instruments that are available in laparoscopic surgery as well^*1*^, but especially the lack of tactile sensation and the reproduction of writs natural movements without a fulcrum. 

Laparoscopic surgery allows physical contact between the hands of the surgeon and the anatomical structure through long and non-flexible instruments^*34*^. Although not perfect, this brings a haptic feedback. This imperfection brings the need for learning. Experient surgeons have increased ability in force control of laparoscopic instruments as compared to novices^*29*^. Oppositely, surgeons and patients are distant in robotic surgery. Some technological advances try to simulate tactile or replace it with other stimuli such as sounds^*2*^, but this is not reality in most systems. Interestingly, the lack of haptic sensation may be compensated with experience^*6*^. The simulator used in this study scores the excessive use of force applied to instruments. We did not analyze mathematically the numbers due to the low statistical power for sub-analysis in a small population, but excessive force use was common in almost all participants from all three groups. 

Different previous studies in simulators showed similar performance in the execution of basic tasks for experienced laparoscopic surgeons and individuals in training (medical students or residents)^18, 21, 23^. The same was observed when experts in open surgery were compared to novices^*5*^ ^,18^. Our results, in concordance with these studies, show some transfer of laparoscopic ability to robotic surgery since experts in laparoscopic surgery performed better than non-experts but in equality to controls. These facts suggest that the robotic platform may understand natural movements allowing controls to perform well and that some laparoscopic abilities (such as inverted movement due to fulcrum) may actually prevent surgeons from performing better than controls forcing to forget some automatic movements to relearn more natural actions. We opted to recruit individuals for the control group that are not linked to health sciences and choose basic not clinical tasks to be executed in order to evaluate natural abilities only. Similarly, we limited age of participants to avoid learned aptitudes with videogames and laic technology. 

### Differences between robotic and laparoscopic learning curves

The learning curve for proficiency seems to be longer for laparoscopic surgery compared to robotic surgery (Table 2) although these studies may be criticized for several reasons: 1) only operative time is considered in most papers, not other parameters such as surgical complications; 2) surgeons with previous experience in the procedure via open or laparoscopy are tested; 3) curve is analyzed after a certain number of cases are operated not based on mathematical calculations; 4) bias of selection of cases for the beginning of experience; 5) expertise is evaluated comparing two periods of time arbitrarily defined; 6) robotic cases are usually more recent; etc. Our study, nonetheless, showed a strong tendency for all groups to learn and perform better even considering only five repetitions of the same task. This fact was also observed by others^*21*^ and it may show a real quick learning characteristic of robotic surgery.

**TABLE 2:** Comparison between learning curves for laparoscopic vs. robotic surgery

Procedure	Laparoscopic surgery	Robotic surgery	References
Esophagectomy	30-40	20-26	13, 15, 32, 37
Gastrectomy	41-46	20-25	14, 17, 38
Roux-em-Y gastric bypass	100-500	8-14	3, 4, 8, 9
Pancreatectomy	15-30	10-40	16, 27, 24, 31
Colectomy	50-85	30-44	12, 22, 25, 35

### Ethics and robotic learning

Simulators are a reality in several residence training programs^*36*^; however, there is an uncountable number of board certified surgeons unfamiliar with robotic surgery. Our protocol evaluated basic manual and coordination skills but, surprisingly, experienced surgeons scored less than 50% of the ideal goal. This shows that simulator training before clinical practice should be mandatory. 

Innovation should be carefully tested before dissemination and it must be followed by adequate training to acquire proficiency^*11*^. Moreover, laboratory training was considered a precondition to consider surgical innovation ethical^*10*^. 

Interestingly, simulators are not only useful for learning. Warming up in simulators brings enhanced performance^*7*^. Following principles of aviation applied to surgery^*30*^, surgeons should keep periodic training in simulators. 

Our study has some limitations such as the small number of participants. The degree of significance of the findings; however, suggests that results were not jeopardized. Also, the tasks we selected may be criticizes. We tried to choose different abilities distant from clinical significance to avoid biases with the control group. The rigorous selection of participants all from the same age is a strong point of the study in our opinion and probably original. 

## CONCLUSION

*E*xperts in laparoscopic surgery performed better than other groups but almost equally to non-medical individuals. Experts in open surgery had worst results. All groups improved performance with repetition. These findings may suggest that robotic surgery reproduce natural movements and it is prone to be quick learned although even experienced laparoscopic surgeons did not perform ideally initially. Surgeons inexperienced in minimally invasive surgery apparently need a longer training. 
